# Supplementary material for: Increase in Double Negative B Lymphocytes in Patients with Systemic Lupus Erythematosus in Remission and Their Correlation with Early Differentiated T Lymphocyte Subpopulations
Source: Curr Issues Mol Biol. 2023 Aug 13;45(8):6667–81. doi: 10.3390/cimb45080421 (PMC10453294; doi:10.3390/cimb45080421)
Supplement: Supplementary file 1 [file cimb-45-00421-s001.zip › Supplement table, figure legends.pdf]

Supplement Table S1. Clinical and laboratory findings of patients with LN at time of diagnosis and time of evaluation

	**SLE patients**	**Healthy controls**
n	30	31
Age (yrs)	43±14	49±13
M/W	1/29	15/16
Time since diagnosis (mo)	84(45-125)	
SLEDAI score	2(1-5)	
SLE manifestations and Systems involved		
• Skin and mucosal involvement	13/30 (43.3%)	
• Musculoskeletal system	7/30 (23.3%)	
• Respiratory system	0/30 (0%)	
• Cardiovascular system	1/30 (3.3%)	
• Haematological disorders	3/30 (10%)	
• Kidney involvement	22/30 (73.3%)	
• Neuropsychiatric disorders	3/30 (10%)	
• Immunological/serological disorders	16/30 (53.3%)	
**Renal Pathology Classification according to ISN/RPS 2003**		
Stage II	3/30 (10%)	
Stage III	3/30 (10%)	
Stage IV	14/30 (46.7%)	
Stage V	7/30 (23.3%)	
Stage III+V	3/30 (10%)	

		CD19IgD-CD27-				CD19IgD-CD27-	
		SLE	HC			SLE	HC
CD4	r	0.548	0.412	CD8	r	0.425	-0.239
	p	0.002	0.021		p	0.019	0.196
Early Differentiated cells							
CD4 CD31+	r	0.587	0.243	CD8 CD31+	r	0.366	-0.286
	p	<0.001	0.188		p	0.047	0.119
CD4 NAÏVE (CD45RA+ CCR7+)	r	0.325	0.189	CD8 NAÏVE (CD45RA+ CCR7+)	r	0.432	-0.308
	p	0.079	0.316		p	0.017	0.091
CD4CD45RA+CD28+	r	0.504	0.265	CD8CD45RA+CD28 +	r	0.234	-0.086
	p	0.005	0.15		p	0.214	0.646
CD4CD45RA+CD57-	r	0.419	0.42	CD8CD45RA+CD57-	r	0.201	-0.251
	p	0.021	0.021		p	0.287	0.173
CD4CD45RA-CD57-	r	0.397	0.24	CD8CD45RA-CD57-	r	0.444	0.166
	p	0.03	0.201		p	0.014	0.371
CD4CD28+CD57-	r	0.487	0.439	CD8CD28+CD57-	r	0.363	-0.088
	p	0.006	0.013		p	0.048	0.638
CD4CD28+CD57+	r	0.491	0.156	CD8CD28+CD57+	r	0.483	-0.092
	p	0.006	0.402		p	0.007	0.624
Memory cells							
CD4 CM (CD45RA-CCR7+)	r	0.381	0.188	CD8 CM (CD45RA-CCR7+)	r	0.33	0.138
	p	0.038	0.32		p	0.075	0.459
CD4 EM (CD45RA-CCR7-)	r	-0.075	0.008	CD8 EM (CD45RA-CCR7-)	r	0.099	0.088
	p	0.695	0.965		p	0.601	0.637
Senescent/Advanced differentiated cells							
CD4 EMRA (CD45RA+ CCR7-)	r	0.104	0.168	CD8 EMRA (CD45RA+CCR7-)	r	0.242	0.04
	p	0.583	0.376		p	0.189	0.182
CD4CD28null	r	0.066	-0.154	CD8CD28null	r	0.271	-0.248

	p	0.73	0.408		p	0.147	0.179
CD4CD28-CD57+	r	- 0.068	-0.243	CD8CD28-CD57+	r	0.291	-0.254
	p	0.722	0.187		p	0.119	0.169
CD4 EMRA (CD45RA+ CCR7-) 28-	r	0.042	0.198	CD8 EMRA (CD45RA+ CCR7-) 28-	r	0.191	-0.032
	p	0.825	0.294		p	0.312	0.862

Supplement Table S2. Correlation of the number of DN B cells with the whole cohort of CD4 and CD8 lymphocytes and their subpopulations.

Supplement Figure Legends

Suppl. Figure S1. Gating strategy for CD4 and CD8 cells, and their subsets, based on the presence of CD45RA, CCR7, CD31, CD57 and CD28

Suppl. Figure S2. Gating strategy for CD19+ cells, and their subsets based on the presence of IgD and CD27
