# Supplementary figures and images for: Increase in Double Negative B Lymphocytes in Patients with Systemic Lupus Erythematosus in Remission and Their Correlation with Early Differentiated T Lymphocyte Subpopulations
Source: Curr Issues Mol Biol. 2023 Aug 13;45(8):6667–81. doi: 10.3390/cimb45080421 (PMC10453294; doi:10.3390/cimb45080421)

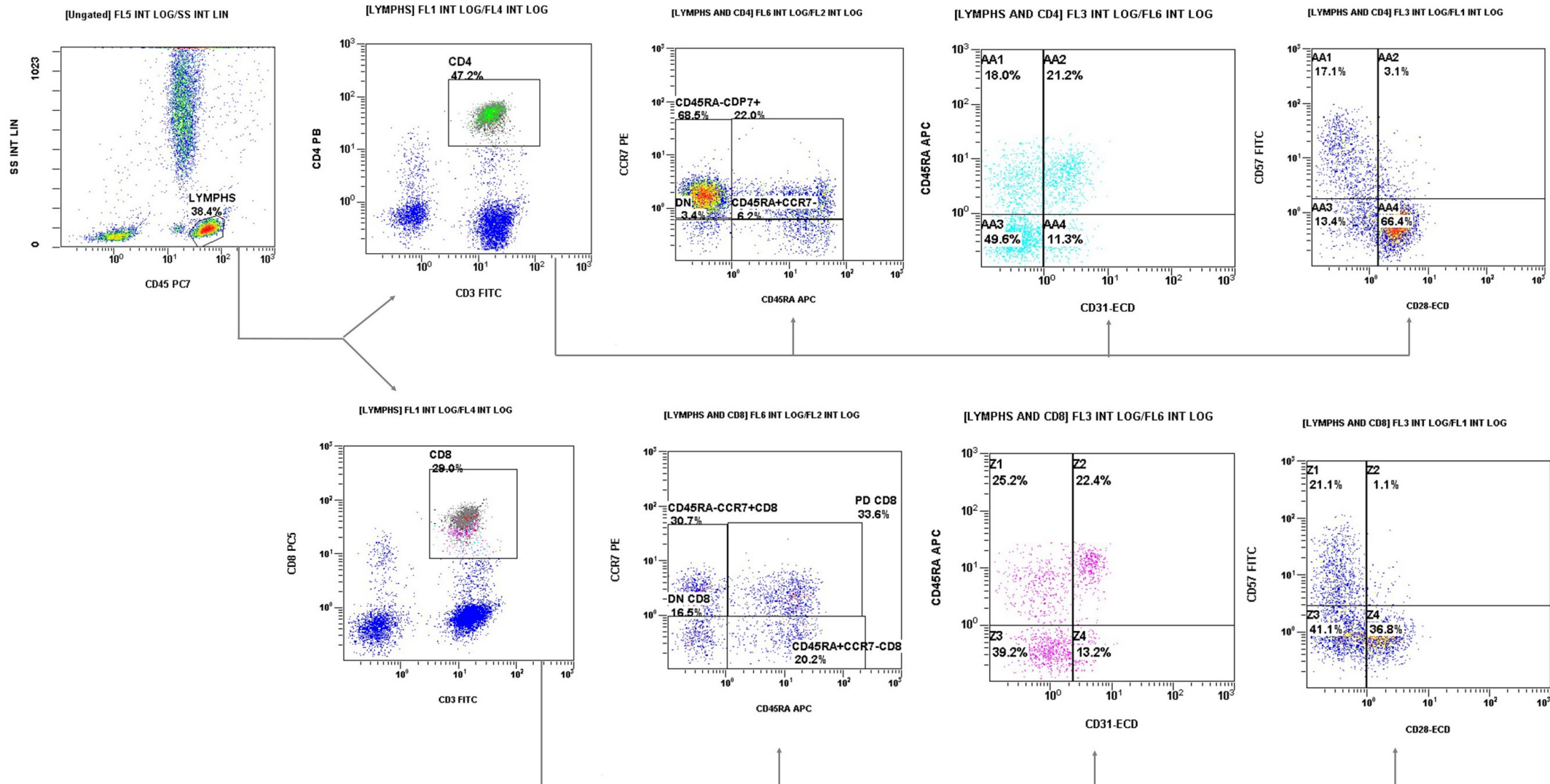

Supplement: Supplementary file 1 [file cimb-45-00421-s001.zip › Suppl Figure S1.pdf]

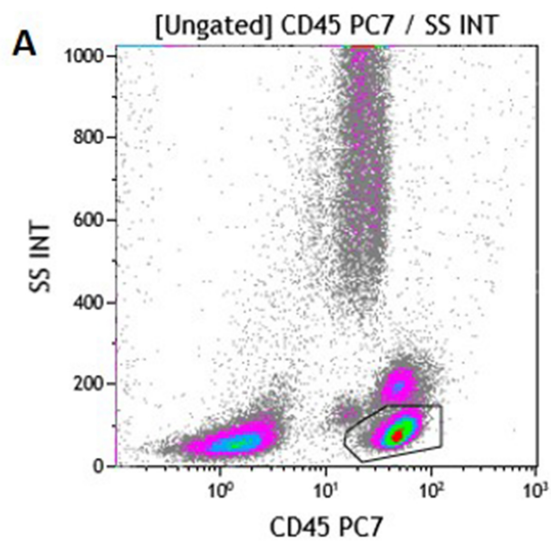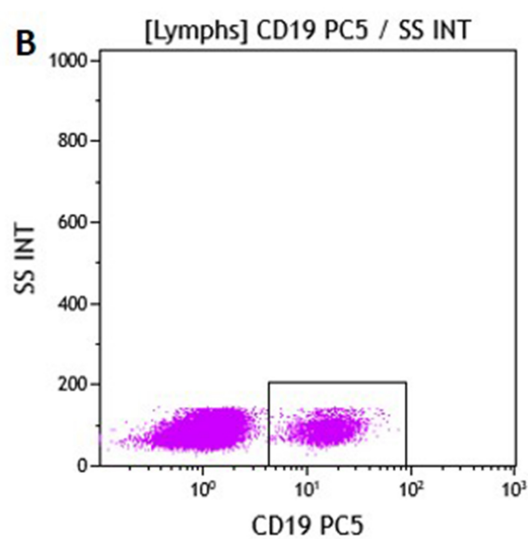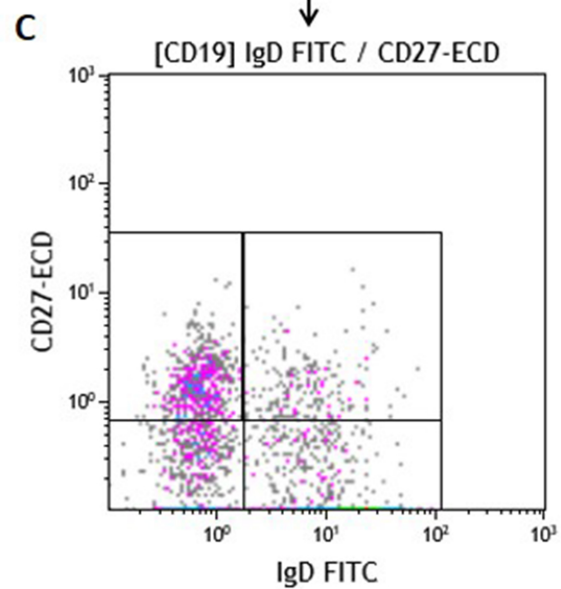

Supplement: Supplementary file 1 [file cimb-45-00421-s001.zip › Suppl Figure S2.pdf]
